# Characteristics of women obtaining induced abortions in selected low- and middle-income countries

**DOI:** 10.1371/journal.pone.0172976

**Published:** 2017-03-29

**Authors:** Sophia Chae, Sheila Desai, Marjorie Crowell, Gilda Sedgh, Susheela Singh

**Affiliations:** Guttmacher Institute, New York, New York, United States of America; Stellenbosch University, SOUTH AFRICA

## Abstract

**Background:**

In 2010–2014, approximately 86% of abortions took place in low- and middle-income countries (LMICs). Although abortion incidence varies minimally across geographical regions, it varies widely by subregion and within countries by subgroups of women. Differential abortion levels stem from variation in the level of unintended pregnancies and in the likelihood that women with unintended pregnancies obtain abortions.

**Objectives:**

To examine the characteristics of women obtaining induced abortions in LMICs.

**Methods:**

We use data from official statistics, population-based surveys, and abortion patient surveys to examine variation in the percentage distribution of abortions and abortion rates by age at abortion, marital status, parity, wealth, education, and residence. We analyze data from five countries in Africa, 13 in Asia, eight in Europe, and two in Latin America and the Caribbean (LAC).

**Results:**

Women across all sociodemographic subgroups obtain abortions. In most countries, women aged 20–29 obtained the highest proportion of abortions, and while adolescents obtained a substantial fraction of abortions, they do not make up a disproportionate share. Region-specific patterns were observed in the distribution of abortions by parity. In many countries, a higher fraction of abortions occurred among women of high socioeconomic status, as measured by wealth status, educational attainment, and urban residence. Due to limited data on marital status, it is unknown whether married or unmarried women make up a larger share of abortions.

**Conclusions:**

These findings help to identify subgroups of women with disproportionate levels of abortion, and can inform policies and programs to reduce the incidence of unintended pregnancies; and in LMICs that have restrictive abortion laws, these findings can also inform policies to minimize the consequences of unsafe abortion and motivate liberalization of abortion laws. Program planners, policymakers, and advocates can use this information to improve access to safe abortion services, postabortion care, and contraceptive services.

## Introduction

Globally, an estimated 56.3 million abortions occurred each year over the period 2010–2014 [1]. This article focuses on low- and middle-income countries (LMICs) (see Data and Methods section for a definition of LMIC), which account for approximately 86% of all abortions [2]. Although little variation exists in abortion incidence across major geographical regions, it does vary widely by subregion and within countries by subgroups of women [3]. Differential abortion levels stem from variation in the level of unintended pregnancies, and variation in the likelihood that women with unintended pregnancies obtain abortions. The incidence of unintended pregnancies, in turn, is determined most immediately by the level of unmet need for contraception and effectiveness of contraceptive use [4, 5]; while variations in seeking abortions could be determined by a range of factors including differences in the opportunity cost of giving birth, strength of motivation to avoid having a child, knowledge of abortion sources, ability to pay for an abortion, and the ease of access to abortion care [5].

Although legal in some LMICs, abortion remains highly restricted in most of Africa and Latin America and the Caribbean (LAC), and parts of Asia [5]. The majority of women in LMICs, aside from China and India where abortion is legal under broad criteria, live in settings with highly restrictive abortion laws [5]. Many of these women obtain abortions in an unsafe manner, raising their risk of abortion-related morbidity and mortality. In such contexts, certain subgroups of women, such as urban and wealthier women, may have better access to safer clandestine abortion services compared to rural and poorer women.

Knowledge of the characteristics of women obtaining abortions could shed light on the subgroups of women especially in need of services to help reduce the incidence and consequences of unintended pregnancies and unsafe abortion. Previous studies have examined the sociodemographic profile of women having abortions in some LMICs [6–8], but in most cases, data are not representative of the cross-section of women obtaining abortions. For example, many country-specific studies are limited in geographical scope, often focusing on a specific city, region/province, or urban/rural area [9–11], or a specific subgroup of women, such as young, unmarried women or university students [12, 13]. In such cases, the study population may not be representative of all women obtaining abortions. Other studies have investigated the characteristics of women who report ever having an abortion, including women who had abortions long before the time of the survey [6, 14, 15]. These findings can be misleading if a woman is classified according to her characteristics at the time of the survey, and these characteristics differ from those at the time of her abortion. The present study addresses the limitations of past studies by analyzing and synthesizing data, collected in large scale surveys over the last decade or so and are in most cases nationally representative, on the characteristics of women obtaining induced abortions in 28 LMICs.

The present study updates findings from a review article on characteristics of women having abortions worldwide, published in the late 1990s [3]. Since this study was published, there has been worldwide change in the demographic and socioeconomic composition of women of reproductive age, attitudes and preferences regarding family formation, and access to reproductive health services, particularly in LMICs. Together, these shifts suggest that the characteristics of women seeking abortions may have also changed. For example, in many countries, age at first marriage has increased and, as a consequence, so have levels of premarital sex [16]. Furthermore, girls are attending school longer and more women are working [17], motivating desires to delay childbearing and have greater control over the timing of births. The increased desire for smaller families and greater control over birth-spacing has been seen particularly in Asia, Europe, and LAC, where desired family size has fallen considerably [18]. These demographic changes may have resulted in more women now considering a pregnancy as unwanted when it might have been considered as wanted twenty years ago. Moreover, women’s access to safe abortion services has grown with increasing liberalization of abortion laws in some countries (e.g. Nepal and Ethiopia) and growing availability of misoprostol in contexts where abortion still remains highly restricted.

This study can help identify which subgroups of women are overrepresented among women having abortion, relative to their population size, and which groups have higher than average abortion rates. We anticipate that study findings will help program planners and policymakers better direct improvements in contraceptive services to those population groups that have the highest levels of abortion. In countries where abortion is highly restricted, we expect that findings will highlight the importance of improving access to postabortion care and safe abortion services, in particular for vulnerable population subgroups.

## Data and methods

This study primarily used nationally representative data, collected between 2002 and 2014, to examine the characteristics of reproductive-age (15–44 years) women obtaining induced abortions in low- and middle-income countries (LMICs). LMICs were defined using the World Bank’s classification of income groups: low-income countries have GNI per capita less than $1,025; lower-middle income countries have GNI per capita between $1,026 and $4,035; and upper-middle income countries have GNI per capita between $4,036 and $12,475 [19]. Twenty-eight countries with economies classified as low-income, lower-middle-income, and upper-middle-income were eligible to be included in this study. Because many factors associated with reproductive behaviors vary by geographic region, we organized results according to major geographical regions: we included five countries from Africa, 13 from Asia, eight from Europe, and two from Latin America and the Caribbean (LAC). All European countries included in this study are located in Eastern or Southern Europe; none are in Western or Northern Europe. Both countries in LAC are located in Central America or the Caribbean. Given that South America is not represented in this study, we referred to this region as Central America and the Caribbean (CAC) for the rest of the article. In addition, for Mexico, we do not have nationally representative abortion data and instead present data for Mexico City, a setting where first-trimester abortion has been legal since 2007 and data are available for the cross-section of women obtaining legal abortions in public sector facilities. Abortion is highly restricted throughout the rest of Mexico. While the study represents a wide range of LMICs, findings should not be generalized to all such countries or entire geographic regions.

### Data sources

We utilized nationally representative data collected in official statistics, population-based surveys, and abortion patient surveys (S1 Table). We analyzed data for all countries except Mexico City (Mexico), for which we extracted information from a peer-reviewed published article [20]. We included abortion data collected from 2000 to 2014; for all countries except Nigeria, Philippines, Uzbekistan, and Vietnam, analyzed data were collected between 2005 and 2014.

Official statistics on legal abortions are available in some LMICs where abortion is permitted under some criteria [21]. Because official statistics are often incomplete or of poor quality in many LMICs [22], we interpreted the data under the assumption that underreporting was random across characteristics. Data for Mexico City are restricted to women who obtained abortions in public sector health facilities; abortions obtained in private sector and NGO facilities and those occurring outside of health facilities were not included.

Population-based surveys such as the Demographic and Health Surveys (DHS) were utilized if they collected abortion data on women of reproductive age. Importantly, population-based surveys generally suffer from high levels of abortion underreporting due to stigma, which could result in large underestimates of abortion incidence [23]. The extent to which underreporting varies according to women’s demographic or socioeconomic characteristics is less well documented. Sensitivity analysis of survey data for one country (Ghana), however, suggests that underreporting does not vary systematically by subgroup, supporting the use of survey data to provide approximate measures of differences between subgroups, despite underreporting [24].

Data for one of our study countries, Ethiopia, came from a survey of patients obtaining abortions in public and private sector health facilities; abortions obtained in NGO sector facilities were not included. These data capture a cross-section of women who have abortions in health facilities. They do not include information collected from women who obtained abortions outside of the formal health sector.

For official statistics and surveys of abortion patients, data were collected for a specific calendar period. For population-based surveys, we used data on abortions that occurred in the three year period before the survey was conducted, unless otherwise specified. We focused on recent abortions because some of the examined characteristics were only measured at the time of the interview. This minimized the possibility that characteristics changed between the time of the abortion and the time of the interview. Still, we expect that for some characteristics, such as marital status and parity, current status would not reflect the situation at the time of the abortion for some women.

For some countries, multiple data sources existed (e.g., official statistics and population-based surveys). In these cases, we selected the data source that we judged to be more representative of the population of women obtaining abortions. We ranked the representativeness of data sources in the following order: official statistics, population-based surveys, and abortion patient surveys. For several countries, we had data from official statistics and population-based surveys and chose to present statistics from the latter source because abortion rates were higher.

Finally, for Ethiopia and Nigeria, we also had access to another source of abortion data: surveys of postabortion care patients. We did not include these data in the main body of the paper because they represent a particular subset of women having abortions—those who experienced complications from unsafe procedures and obtained postabortion care—and likely do not represent the cross-section of all women having abortions. In S2 Table, we include data from this source and another, more representative source, as a valuable comparison.

### Abortion measures

We calculated the percentage distribution of abortions by women’s characteristics for all countries in our study. Where the proportion of abortions matches the proportion of women of reproductive age across population subgroups, we infer that these subgroups do not disproportionately contribute to abortions.

Abortion rates were calculated as the number of abortions for every 1,000 women of reproductive age (15–44 years). For abortion rates calculated using population-based data, denominators were calculated as person-years contributed to the sample during the three-year period before the survey, unless otherwise indicated. In contrast to the percentage distribution, abortion rates account for the size of subgroups and thus better represent subgroup differences in the likelihood a woman obtains an abortion. Abortion rates have been included in S3 and S4 Tables and, where appropriate, are discussed in the text. Because underreporting is expected in nearly all of the evidence presented, the rates should be interpreted as minimum estimates and not accurate measures of abortion incidence.

### Women’s characteristics

We examined abortion differentials by age at abortion, marital status, parity, wealth, education, and residence. Marital status was categorized as married and unmarried; women were coded as married if they reported being married, in union, or cohabiting, and unmarried if they reported being divorced, separated, widowed, or never-married. In population-based surveys of Asian countries where all women were interviewed, regardless of marital status, we observed very few abortions among unmarried women (never married, separated, divorced, and widowed), even though they comprised a sizeable fraction of all interviewed women. Due to concerns of abortion underreporting by unmarried women, we restricted analyses to currently married women in these countries. For one of our sources, the DHS surveys, a composite measure of household wealth was available and calculated based on household ownership of selected assets using principal components analysis [25]. We present results according to wealth quintiles of households in which women reside (poorest, second, middle, fourth, and richest).

### Analyses

We used STATA version 14 (StataCorp LP, College Station, TX) for all analyses and utilized survey-specific sample weights to construct representative estimates. We report all results by region and country. Age-specific findings for women aged 15–19 years and 40–44 years include abortions that occurred among women younger than 15 years and older than 44 years, respectively.

## Results

### Age at abortion

In four of the five African countries covered in this article, approximately one-quarter of reported abortions occurred among adolescent women aged 15–19 (Table 1). In these countries, adolescents make up 22–26% of reproductive-age women (S5 Table), indicating that adolescents do not disproportionally obtain abortions. Nigeria is the one exception where over one-third of abortions took place among adolescents. In all African countries except Nigeria, women aged 20–29 accounted for more than half of all reported abortions, despite making up only 40% of reproductive-age women. In contrast, 11–23% of reported abortions in this region occurred among women in their thirties and only 1–4% among women aged 40–44. These figures are disproportionately low compared to the proportion of women 30 years and older. Age-specific abortion rates for countries with these data generally reflect inferences made from the percentage distribution of abortions (S3 Table). In Congo Republic and Gabon, abortion rates were highest among women in their twenties, while in Nigeria and Ghana, they were highest among women aged 15–19 and 20–24, respectively.

**Table 1:** Percentage distribution of abortions by age group, by region and country.

	15–19	20–24	25–29	30–34	35–39	40–44	Number of abortions		15–19	20–29	30–39	40–44
*Africa*												
Congo Republic, 2011–12^a^	21.4	32.8	23.2	12.8	7.6	2.3	772		21	56	20	2
Ethiopia, 2014^b^	28.7	36.8	22.7	7.9	3.3	0.5	2,615		29	60	11	1
Gabon, 2012^a^	20.1	30.5	23.2	15.2	7.4	3.6	542		20	54	23	4
Ghana, 2007	24.2	32.8	19.8	11.9	8.8	2.5	399		24	53	21	3
Nigeria, 2002–03^a^	34.2	19.7	21.2	11.0	9.6	4.2	205		34	41	21	4
*Asia*^*c*^												
Armenia, 2010	1.9	22.8	37.7	23.7	8.4	5.5	395		2	61	32	6
Azerbaijan, 2006	1.8	18.7	27.1	26.5	17.8	8.0	1,504		2	46	44	8
Bangladesh, 2011^d^^,^^e^	6.0	20.4	28.6	21.4	15.9	7.8	381		6	49	37	8
Cambodia, 2010^e^^,^^f^	1.1	12.6	21.4	22.8	21.2	20.9	1,070		1	34	44	21
Georgia, 2011^g^	5.5	49.2^i^	-	28.0	12.8	4.4	30,590		6	49	41	4
Kyrgyz Republic, 2012	1.1	21.7	34.4	21.1	14.0	7.7	427		1	56	35	8
Nepal, 2011	6.2	19.2	34.5	25.9	12.0	2.2	341		6	54	38	2
Pakistan, 2012–13	5.6	13.0	31.3	31.7	11.5	7.0	115		6	44	43	7
Philippines, 2004^a^	6.5	20.9	24.9	21.5	16.6	9.5	193		7	46	38	10
Tajikistan, 2012	1.3	17.4	26.8	22.3	23.2	9.0	365		1	44	46	9
Turkey, 2008	2.3	9.6	22.6	27.1	26.3	12.2	339		2	32	53	12
Uzbekistan, 2002	0.9	18.4	26.4	24.9	17.8	11.6	366		1	45	43	12
Vietnam, 2002	0.2	11.2	29.4	22.9	23.7	12.6	403		0	41	47	13
*Europe*												
Albania, 2008–09	5.4	16.6	24.2	26.0	19.2	8.6	143		5	41	45	9
Belarus, 2013^g^	6.0	19.3	26.9	25.3	15.3	7.2	31,206		6	46	41	7
Bulgaria, 2013^g^	8.8	20.3	24.5	22.9	17.9	5.7	29,505		9	45	41	6
Moldova, 2005	5.3	27.0	26.2	22.5	14.2	4.6	607		5	53	37	5
Montenegro, 2005^g^	2.8	35.2^i^	-	50.4^j^	-	11.7	1,948		3	35	50	12
Romania, 2012^g^	9.1	21.6	23.0	22.3	16.8	7.2	87,975		9	45	39	7
Serbia, 2008^g^	4.3	15.0	22.4	26.0	21.6	10.7	22,866		4	37	48	11
Ukraine, 2007	6.1	21.4	31.2	21.4	13.6	6.2	278		6	53	35	6
*Central America & the Caribbean*												
Haiti (2012)^a^	13.8	28.8	23.6	15.2	13.1	5.5	202		14	52	28	6
Mexico City (Mexico), 2007–2010^h^	17.1	36.0	22.1	13.9	8.2	2.8	20,053		17	58	22	3
								**AVG**	**15**	**55**	**25**	**4**

Note: All data are from population-based surveys unless otherwise specified. Calculations are based on all abortions reported in the three year period before the survey unless otherwise noted.

^a^ Calculations based on the most recent reported abortion in the three year period before the survey.

^b^ Calculations based on abortions obtained in the year(s) of data collection. Data are from a survey of patients who obtained abortions in public and private sector health facilities. Abortions obtained in NGO affiliated health facilities are not included.

^c^ Calculations based on samples of currently married women. For Georgia, calculations based on all women.

^d^ Menstrual regulation used as proxy for abortion.

^e^ Calculations based on age at the time of survey.

^f^ Calculations based on reported abortions in the five year period before the survey.

^g^ Data are from official statistics on legal abortion.

^h^ Calculations based on abortions obtained in the year(s) of data collection. Data are from a survey of abortion patients who obtained abortions in public sector health facilities. Abortions obtained in private sector facilities are not included.

^i^ Calculation based on women 20–29 years.

^j^ Calculation based on women 30–39 years.

Data for 12 of the 13 Asian countries in our study were collected from currently married women. On average, 3% of abortions occurred to married adolescents in all countries except Georgia. Abortions disproportionately occurred among married women aged 20–29; despite making up 40% of reproductive-age women, 46% of abortions occurred among married women in this age range. Approximately 42% of abortions occurred to married women aged 30–39, which is slightly higher than the percentage of reproductive-age women in this age range. Compared to the African countries in our study, the Asian countries had a substantially higher percentage of reported abortions (7–21% in 10 of the 12 countries) among married women aged 40–44. Although data for Georgia included abortions to all women, regardless of marital status, the distribution of abortions followed a similar age pattern to that of currently married women in the other 12 Asian countries in our study. In most Asian countries, abortion rates were highest among women aged 25–29 and 30–34 (S4 Table). In Armenia and the Philippines, abortion rates were also high among women aged 20–24, and in Cambodia, they were high among women aged 35–39. In Georgia, the abortion rate was highest among women aged 20–29 (the data was available only for the ten-year age-range) and women aged 30–34.

The eight European countries in our sample followed a similar pattern to the Asian countries. On average, only 6% of abortions occurred among adolescents, even though 15–19 year olds accounted for approximately 16% of reproductive-age women in those countries. Women in their twenties, on the other hand, made up a disproportionate share of abortions, contributing to 44% of abortions. Women in their thirties accounted for 35–50% of abortions, which is slightly higher than the percentage of reproductive-age women in this age range. Despite making up 16–20% of the reproductive age population, only 5–12% of abortions occurred to women aged 40–44. In most countries, abortion rates were highest among 25–29 year olds, with similar or slightly lower rates among women aged 20–24 and 30–34 (S3 Table).

In Haiti, women aged 15–19 accounted for 26% of all reproductive-age women but only 14% of abortions. In Mexico City, in contrast, adolescent women contributed to only 17% of abortions. In both settings, women aged 20–29 accounted for the majority of abortions, despite making up only 37% of the population of reproductive-age women. Approximately one-quarter of abortions occurred among women aged 30–39, and 3% of abortions in Mexico City and 6% in Haiti among women aged 40–44.

### Marital status

**Fig 1:**
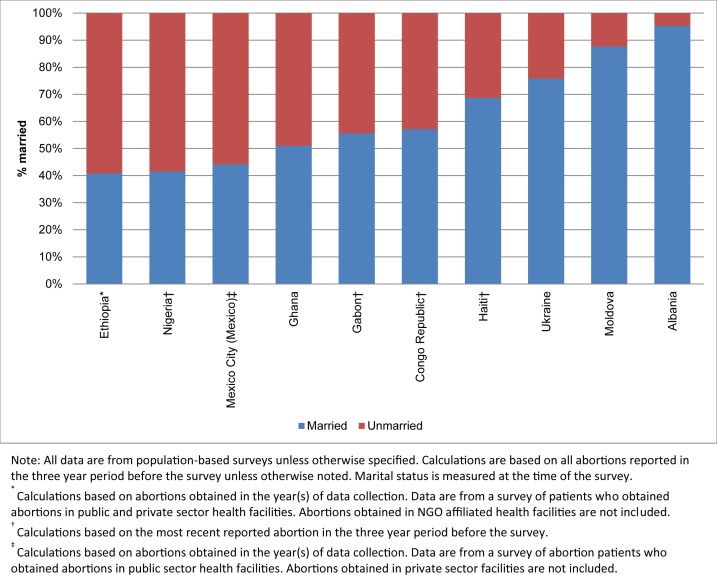
Percentage distribution of abortions by marital status.

Given that data on abortions by marital status were only available for 10 settings, none of which are located in Asia, we do not present the data by region (Fig 1). In seven of the ten countries (Ghana, Gabon, Congo Republic, Haiti, Ukraine, Moldova, and Albania), the majority of abortions were among married women. In three of the settings (Ethiopia, Nigeria, and Mexico City), unmarried women accounted for a disproportionate share. Abortion rates by marital status were available for eight countries (S3 Table). In Albania, Moldova, Ukraine, and Haiti, abortion rates were higher among married women, while in Gabon, they did not differ by marital status. In Congo, Ghana, and Nigeria, unmarried women had higher abortion rates.

### Parity

**Fig 2:**
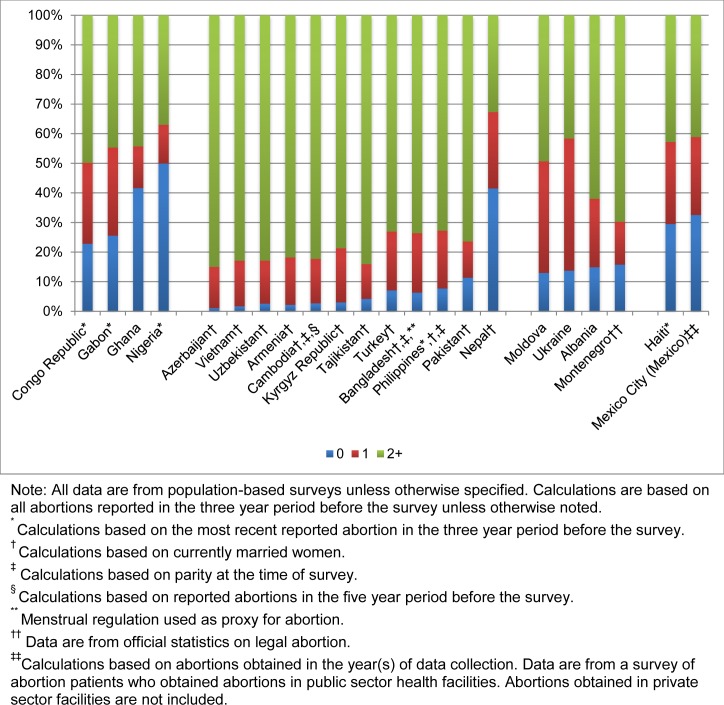
Percentage distribution of abortions by parity.

Among the African countries, variation exists in the distribution of abortions by parity (Fig 2). Almost half of all abortions occurred among nulliparous women in Ghana and Nigeria. Nulliparous women comprise approximately 33% of reproductive-age women in these countries (S5 Table). In contrast, only a quarter of abortions occurred among nulliparous women in Congo Republic and Gabon. Instead, disproportionate fractions of abortions, 27% and 30%, occurred among women who had one birth in Congo Republic and Gabon, respectively. In all four African countries, multiparous women (those who have had two or more births) accounted for approximately 44% of abortions, which is slightly lower than the percentage of reproductive-age women who are multiparous. In Congo Republic and Gabon, abortion rates were highest among women who had one birth, while nulliparous women in Ghana and Nigeria had the highest abortion rates (S3 Table).

In 11 of the 12 Asian countries with evidence (based on married women), the majority of abortions (73–85%) occurred among multiparous women (Fig 2). In most of these countries, multiparous women accounted for a somewhat disproportionately high share of reported abortions (their share of all married women aged 15–44 ranges from 67% to 77% in S5 Table). The pattern was different in Nepal: A much smaller proportion (one-third of abortions) occurred among multiparous women, while 42% occurred among nulliparous women. In all countries except Nepal and the Philippines, abortion rates were highest among women who had two or more births (S4 Table). In Nepal and the Philippines, nulliparous women had the highest abortion rates.

In the four European countries for which we have data, nulliparous women contributed to 13–16% of abortions. In Ukraine, women with one previous birth made up almost half of all abortions while multiparous women in Albania, Moldova, and Montenegro accounted for 49–70%. In Albania and Moldova, abortion rates were highest among women who had one birth, and in Ukraine, they were highest among multiparous women.

Evidence on parity of women obtaining abortions was available for both CAC countries. Abortions were distributed relatively evenly across parity categories, with slightly higher proportions among multiparous women. Data on the underlying distribution of women by parity were available only for Haiti: 43% were nulliparous; 18% had one birth; and 38% were multiparous. These data suggest that women who had one birth (28% of all abortions among women aged 15–44) accounted for a disproportionate share of abortions.

### Wealth

Of the 28 countries covered in this study, 19 countries had information on household wealth. Results showed that wealthier women (top two quintiles) were disproportionately more likely to report having an abortion in 12 countries (three in Africa, seven in Asia, one in Europe, and one in CAC) (Table 2). In Armenia and Azerbaijan, a higher proportion of abortions occurred among women in the poorest wealth quintile. In the remaining countries (Albania, Ukraine, Nigeria, the Philippines, and Vietnam), no pattern or a much weaker pattern was observed by wealth quintile. Abortion rates (S3 and S4 Tables) generally follow a similar pattern to that of the percentage distribution of abortions.

**Table 2:** Percentage distribution of abortions by wealth, by region and country.

	Poorest	Second	Middle	Fourth	Richest	Number of abortions
*Africa*						
Congo Republic^a^	8.0	19.1	23.2	25.4	24.3	772
Gabon^a^	14.5	21.5	21.2	22.5	20.3	542
Ghana	7.5	6.9	20.4	30.1	35.0	399
Nigeria^a^	20.2	26.7	15.2	19.6	18.3	205
*Asia*^b^						
Armenia	26.6	16.3	26.5	14.5	16.0	395
Azerbaijan	25.3	15.8	22.9	17.5	18.4	1,504
Bangladesh^c^	10.7	16.2	16.3	22.5	34.3	381
Cambodia^d^	17.3	18.1	17.3	25.5	21.8	1,070
Kyrgyz Republic	13.9	16.7	15.5	28.0	25.9	427
Nepal	10.7	13.3	16.0	23.2	36.9	341
Pakistan	3.8	12.2	17.6	28.4	37.9	115
Philippines^a^^,^^e^	33.6	-	37.0	-	29.4	193
Tajikistan	16.9	14.5	17.9	23.9	26.9	365
Turkey	11.2	20.0	22.2	14.9	31.7	339
Vietnam	20.9	17.0	22.6	22.2	17.3	403
*Europe*						
Albania	17.5	21.3	27.5	19.7	13.9	143
Moldova	10.4	17.0	17.8	23.1	31.8	607
Ukraine	18.3	25.0	19.7	17.0	20.0	278
*Central America & the Caribbean*						
Haiti^a^	1.0	3.5	15.5	35.1	44.8	202

Note: All data are from population-based surveys. Unless otherwise noted, calculations are based on all abortions reported in the three year period before the survey. Wealth is measured at the time of the survey.

^a^ Calculations based on the most recent abortion in the three year period before the survey.

^b^ Calculations based on sample of currently married women.

^c^ Menstrual regulation used as proxy for abortion.

^d^ Calculations based on abortions in the five year period before the survey.

^e^ Wealth categories are lowest, middle, and richest.

### Educational attainment

**Fig 3:**
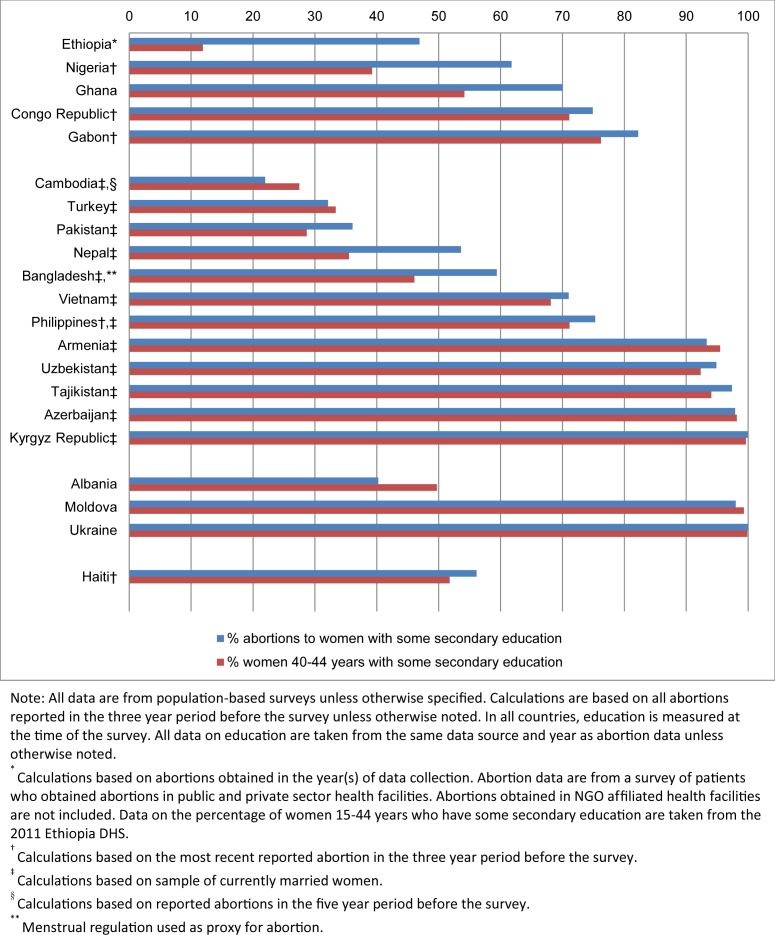
Percent of abortions to women with some secondary education and percent of women with some secondary education.

In four of the five African countries with education data, women with higher levels of education (at least one year of secondary education or more) accounted for the majority of abortions (Fig 3); this ranged from 61% in Nigeria to 82% in Gabon. With the exception of Congo Republic and Gabon, all African countries reported a disproportionate share of abortions among women with some secondary education. Abortion rates were higher among more educated women in the four African countries where abortion rates could be calculated (S3 Table).

In the 12 Asian countries with education data, the percentage of abortions occurring among women with some secondary education ranged from 22% in Cambodia to 100% in Kyrgyz Republic. This variation is primarily due to differences in the underlying distribution of the population by educational attainment. In eight of the 12 Asian countries in our study, the distribution of abortions by education was similar to the underlying distribution of women by education. In three countries (Pakistan, Nepal and Bangladesh), women with secondary education were disproportionately represented among women having abortions. Cambodia was the only exception where a smaller proportion of abortions occurred among women with at least some secondary education (22%) compared to the proportion of women with secondary education (28%). Abortion rates were higher among more educated women in seven of the 12 Asian countries with these data (S4 Table).

Only three of the eight European countries in our study had data on education. In Moldova and Ukraine, the distribution of abortions was similar to the underlying distribution of women by education. In Albania, a smaller proportion of abortions occurred among women with at least some secondary education compared to the proportion with secondary education.

In Haiti, the proportion of women with at least some secondary education was similar to the proportion of abortions among this subgroup of women; abortion rates were also similar by educational attainment (S3 Table).

### Rural/Urban residence

**Fig 4:**
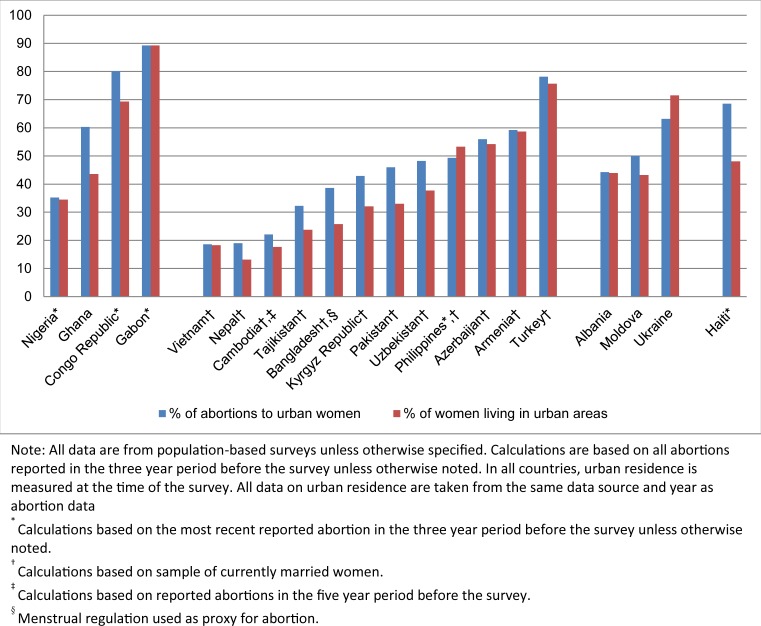
Percent of abortions to urban women and percent of women living in urban areas.

In all African countries, except Nigeria, the majority (60–89%) of reported abortions occurred among urban women (Fig 4). In Gabon, the percentage (89%) of abortions among urban women matched the underlying distribution of women. In Ghana and Congo Republic, the data suggest that a disproportionate share of abortions occurred among urban women. In Nigeria, 35% of reported abortions occurred among urban women, a figure which matches the underlying distribution of women. Similar patterns were found in abortion rates (S3 Table).

In the Asian countries, the percentage of abortions reported by urban women ranged from 19% in Vietnam to 78% in Turkey. In many of these countries, the proportions were comparable to the underlying distribution of women living in urban areas. However, in Bangladesh, Kyrgyz Republic, Pakistan, and Uzbekistan, urban women were disproportionately more likely to have abortions (a difference of ten or more percentage points between the proportion of abortions and the proportion of women living in urban areas). We calculated abortion rates for most of the Asian countries covered in this study (S4 Table). We observed higher abortion rates in urban areas, with the exception of Armenia, Azerbaijan, the Philippines, and Vietnam, where rates for urban and rural women are similar.

In Albania and Moldova, 44–50% of abortions were reported to have occurred among urban women, a proportion which is roughly similar to the underlying distribution of women by residence. While 72% of women in Ukraine live in urban areas, they account for only 63% of abortions.

In Haiti, the only CAC country with residence data, the majority (69%) of abortions occurred among women living in urban areas. This is higher than the proportion of women living in urban areas (48%), and this difference is reflected in the higher abortion rates occurring among urban women (S3 Table).

## Discussion

Despite the demographic changes that have occurred in LMICs, the characteristics of women obtaining abortions have remained largely unchanged. Consistent with findings from a review article from the late 1990s [3], we demonstrate that women across all demographic and socioeconomic subgroups obtain abortions. Across the four regions, many similarities exist in the characteristics of women obtaining abortions, yet there are notable differences between and within regions. We discuss some of the interesting patterns observed in the profiles of these women.

In many LMICs, rising ages at first marriage combined with increasing levels of premarital sex [16] continue to result in unintended pregnancies among adolescents. In addition, adolescent girls are remaining in school longer, which may factor into their decisions to postpone childbearing and terminate unintended pregnancies [26]. A review from the late 1990s found that young women often cited a desire to stay in school as a major reason for abortion [27]. Moreover, many adolescents continue to have difficulty accessing contraception, often as a result of stigma and/or lack of resources [28–30]. This could explain why a substantial proportion of abortions were concentrated among young, unmarried, and nulliparous women in many of the African countries in our study. We observed this pattern, but to a lesser extent, in the European and CAC countries, where proportions were lower but still substantial among adolescents. In the Asian countries, though, data are inadequate to assess this pattern. Due partly to lack of data and partly to concerns of abortion underreporting by unmarried women, our study restricted analyses to currently married women in this region. Thus, we cannot determine if young, unmarried, and nulliparous women make up a sizeable fraction of abortions in the Asian countries included in this study.

Increased desire for smaller families and greater control over birth spacing has been seen, particularly in Europe, Asia, and CAC, where fertility rates have fallen considerably over the past three or more decades. In the majority of Asian countries, a disproportionate share of abortions occurred among multiparous married women, suggesting that these women often obtain abortions to space or limit births in order to have smaller families. This finding likely extends to other LMICs, too, where desires for smaller families may motivate some women to obtain abortions. Furthermore, in many of these countries, unmet need for contraception continues to be high; for example, in Western Asia, of women who would like to delay or limit their childbearing, 50% were not using a modern contraceptive method [4]. If effective contraceptive use does not increase to meet changing fertility preferences, the number of unintended pregnancies (and abortions) will likely rise. In addition, without adequate access to safe abortion services, some women will resort to unsafe abortion and face preventable abortion-related morbidity and mortality.

Not surprisingly, in developing countries across all four regions, a disproportionate share of abortions occurred to women in their twenties. Women in this age range are likely to be married, sexually active, and fecund. In contrast to younger and older women who may want to delay, space, or limit their births, women in their twenties may terminate unintended pregnancies for any of these reasons. In contrast to findings from countries in Africa, we found that a sizeable proportion of reported abortions in Asian and European countries occurred among older women, further suggesting that women in these countries obtain abortions to limit births.

Our study also found that, in some countries, a disproportionate share of abortions occurred among women of high socioeconomic status (SES), as measured by urban residence, educational attainment, and wealth status. Desired family size usually declines faster in urban areas and among more educated and wealthier women, which is one explanation why abortions might be occurring disproportionately among women in these subgroups. If contraceptive use lags behind women’s increasing desires to prevent unintended pregnancy and control the timing of births, women may turn to abortion as a way of controlling their fertility. Women of high SES may also experience a disproportionate share of abortions because they have better access to information on where to obtain safe abortion services, more empowerment to act on fertility preferences, and greater strength and motivation to avoid unplanned births compared to their counterparts of lower SES.

In exploratory analyses, we organized countries by economic development based on income level and restrictiveness of abortion laws. We found no obvious patterns in the data, and instead observed that regional patterns prevailed. This may be expected given that countries within a region often share similar cultures, which could influence marriage patterns, sexual behavior, fertility desires, and contraceptive use. Furthermore, among LMICs, income level may not be the best indicator of development. Despite high income levels, some countries may still measure poorly on other important indicators of development such as education and life expectancy. In addition, abortion laws and their restrictiveness tend to be similar within regions. Abortion laws are more restrictive in African and CAC countries, and less restrictive in the Asian and European countries for which we have data.

This study contains several limitations. The geographic representation of this study is limited by the availability of abortion data. Several subregions with LMICs are not represented, including Northern Africa, Southern Africa, and South America. Data availability was especially limited in LAC. Moreover, two of the most populous LMICs, China and India, are not represented in this study. As a result, we are not able to make broad generalizations about the characteristics of women obtaining abortions in LMICs.

For 13 countries in this study, we analyzed data collected in the early to late 2000s. Specifically, data for four countries (Nigeria, Philippines, Uzbekistan, Vietnam) were collected before 2005 and data for nine (Ghana, Azerbaijan, Turkey, Albania, Moldova, Montenegro, Serbia, Ukraine, and Mexico City) were collected in 2005–2009. Though some LMICs have experienced changes in the population distribution of key characteristics, we observed only small changes in the distribution of women according to age, marital status, and urban residence in the four countries with data from the early 2000s [31, 32]. Significant changes in levels of contraceptive use and unmet need that differ according to women’s characteristics could also differentially affect the rate of unintended pregnancy and the likelihood that women obtain abortions, in different population subgroups. Although potentially dated, these data provide hard-to-find, valuable information on abortions and the characteristics of women obtaining abortions in LMICs. Nevertheless, program planners and policymakers should exercise caution before generalizing from these findings.

Women reporting abortions may not be representative of all women who obtained abortions. For example, differential underreporting of abortions by marital status may result in an underestimation of the proportion of abortions occurring to unmarried women. Due to cultural stigma related to premarital sex, unmarried women may be more likely to underreport abortions. Underreporting may also be more common among younger, nulliparous, and possibly more educated women, given that unmarried women disproportionately comprise these subgroups. We examined whether differential underreporting of abortions exists by marital status in Moldova and the Ukraine by comparing the age distribution of abortions calculated using DHS data with official statistics (S6 Table). Compared to DHS data, official statistics showed that a higher percentage of abortions occurred among adolescents. Given that very few adolescents are married in these countries, it is highly probable that unmarried women are underreporting abortions.

Differential underreporting of abortions by marital status is a larger issue in the Asian countries in our study, because stigma against sexual activity among unmarried women is stronger in conservative societies. As a result, we restricted all analyses for the Asian countries to currently married women, excluding Georgia, where abortions to all women were included. However, focusing on abortion among only currently married women is an important limitation, especially in contexts where a substantial share of abortions occur to unmarried women. For three of the Asian countries in our study (Azerbaijan, Kyrgyz Republic, and Tajikistan), we compared the age distribution of abortions calculated using DHS data with official statistics (S6 Table). While DHS data showed that 1–2% of reported abortions occurred among adolescent women, official statistics indicated that 5–9% of abortions occurred among women in this age range. While these data suggest that some unmarried women are obtaining abortions, they constitute a very small minority and it is likely that married women account for the majority of abortions [1].

Furthermore, we used the percentage distribution of abortions across characteristics as our main abortion measure. Percentage distributions, however, may be biased if differential underreporting exists across the population subgroups. For example, due to stigma, younger and unmarried women may be more likely to underreport abortions compared to older and married women. This problem, however, might be minimal as evidence in at least one context suggests that underreporting does not vary systematically by subgroup [24]. This measure also has limitations with respect to subgroup comparisons across countries, should the distribution of underlying populations of reproductive-age women vary substantially across countries. Nevertheless, these findings on the profile of women obtaining abortions can be of program and policy relevance.

## Conclusion

In many LMICs, abortion is illegal or highly restricted, leading some women to seek unsafe abortions. As a result, population subgroups with disproportionate levels of abortion are also likely to suffer from greater health, social, and economic consequences of unsafe abortion. For example, each year, millions of women in LMICs obtain treatment in health facilities for complications from unsafe abortions [33] and thousands of women die as a result [34, 35]. In these settings, improved access to postabortion care is needed—for all women, but especially for subgroups that have higher levels of abortion and/or experience greater socioeconomic inequity.

Our study takes a first step in synthesizing available evidence on the characteristics of women obtaining abortions in 28 LMICs. Abortion is highly legally restricted in about half of these countries, and broadly legal in the rest of these countries, though safe abortion services are not universally accessible in some of the latter group of countries. Our findings suggest which subgroups of women are at highest risk of unintended pregnancies in all of the 28 study countries and which are therefore in greatest need of better contraceptive information and services. Our findings also suggest which subgroups are likely to have the highest levels of unsafe abortion (in countries where access to safe abortion is highly restricted by law): these subgroups are also in need of improved access to quality postabortion care services. In addition, advocacy to improve access to safe and legal abortion services is needed, particularly in countries with highly restrictive laws but also in countries where it is broadly legal but where some subgroups have inadequate access to safe abortion services.

This study also demonstrates a need for more widespread data collection; relevant data were only available for 28 of 83 LMICs. Nevertheless, currently available data presented in this article will help program planners and policymakers improve contraceptive services and postabortion care, and expand access to safe abortion services in more countries. Doing so would help more women have greater control over their fertility and achieve the family size they desire, and it would reduce abortion-related morbidity and mortality.

## Supporting information

S1 Table List of sources of data. (PDF)

S2 Table Characteristics of women obtaining abortions in Ethiopia and Nigeria by data source. (PDF)

S3 Table Abortion rates (number of abortions per 1000 women ages 15–44) by country in Africa, Europe, and Central America and the Caribbean. (PDF)

S4 Table Abortion rates (number of abortions per 1000 women ages 15–44) by country in Asia. (PDF)

S5 Table Percentage distribution of reproductive-age women by sociodemographic characteristics, by region and country. (PDF)

S6 Table Percentage distribution of abortions by age and data source. (PDF)
