# Correction: Characteristics of women obtaining induced abortions in selected low- and middle-income countries

**DOI:** 10.1371/journal.pone.0177149

**Published:** 2017-05-03

**Authors:** Sophia Chae, Sheila Desai, Marjorie Crowell, Gilda Sedgh, Susheela Singh

In Table 1, the five columns, “15–19”, “20–29”, “30–39”, and “40–44”, are incorrectly duplicated to the right of the “Number of abortions” column. The last column should be “Number of abortions”.

**Table 1:** Percentage distribution of abortions by age group, by region and country.

	15–19	20–24	25–29	30–34	35–39	40–44	Number of abortions
*Africa*							
Congo Republic, 2011–12^a^	21.4	32.8	23.2	12.8	7.6	2.3	772
Ethiopia, 2014^b^	28.7	36.8	22.7	7.9	3.3	0.5	2,615
Gabon, 2012^a^	20.1	30.5	23.2	15.2	7.4	3.6	542
Ghana, 2007	24.2	32.8	19.8	11.9	8.8	2.5	399
Nigeria, 2002–03^a^	34.2	19.7	21.2	11.0	9.6	4.2	205
*Asia*^*c*^							
Armenia, 2010	1.9	22.8	37.7	23.7	8.4	5.5	395
Azerbaijan, 2006	1.8	18.7	27.1	26.5	17.8	8.0	1,504
Bangladesh, 2011^d^^,^^e^	6.0	20.4	28.6	21.4	15.9	7.8	381
Cambodia, 2010^e^^,^^f^	1.1	12.6	21.4	22.8	21.2	20.9	1,070
Georgia, 2011^g^	5.5	49.2^i^	-	28.0	12.8	4.4	30,590
Kyrgyz Republic, 2012	1.1	21.7	34.4	21.1	14.0	7.7	427
Nepal, 2011	6.2	19.2	34.5	25.9	12.0	2.2	341
Pakistan, 2012–13	5.6	13.0	31.3	31.7	11.5	7.0	115
Philippines, 2004^a^	6.5	20.9	24.9	21.5	16.6	9.5	193
Tajikistan, 2012	1.3	17.4	26.8	22.3	23.2	9.0	365
Turkey, 2008	2.3	9.6	22.6	27.1	26.3	12.2	339
Uzbekistan, 2002	0.9	18.4	26.4	24.9	17.8	11.6	366
Vietnam, 2002	0.2	11.2	29.4	22.9	23.7	12.6	403
*Europe*							
Albania, 2008–09	5.4	16.6	24.2	26.0	19.2	8.6	143
Belarus, 2013^g^	6.0	19.3	26.9	25.3	15.3	7.2	31,206
Bulgaria, 2013^g^	8.8	20.3	24.5	22.9	17.9	5.7	29,505
Moldova, 2005	5.3	27.0	26.2	22.5	14.2	4.6	607
Montenegro, 2005^g^	2.8	35.2^i^	-	50.4^j^	-	11.7	1,948
Romania, 2012^g^	9.1	21.6	23.0	22.3	16.8	7.2	87,975
Serbia, 2008^g^	4.3	15.0	22.4	26.0	21.6	10.7	22,866
Ukraine, 2007	6.1	21.4	31.2	21.4	13.6	6.2	278
*Central America & the Caribbean*							
Haiti (2012)^a^	13.8	28.8	23.6	15.2	13.1	5.5	202
Mexico City (Mexico), 2007–2010^h^	17.1	36.0	22.1	13.9	8.2	2.8	20,053

Note: All data are from population-based surveys unless otherwise specified. Calculations are based on all abortions reported in the three year period before the survey unless otherwise noted.

^a^ Calculations based on the most recent reported abortion in the three year period before the survey.

^b^ Calculations based on abortions obtained in the year(s) of data collection. Data are from a survey of patients who obtained abortions in public and private sector health facilities. Abortions obtained in NGO affiliated health facilities are not included.

^c^ Calculations based on samples of currently married women. For Georgia, calculations based on all women.

^d^ Menstrual regulation used as proxy for abortion.

^e^ Calculations based on age at the time of survey.

^f^ Calculations based on reported abortions in the five year period before the survey.

^g^ Data are from official statistics on legal abortion.

^h^ Calculations based on abortions obtained in the year(s) of data collection. Data are from a survey of abortion patients who obtained abortions in public sector health facilities. Abortions obtained in private sector facilities are not included.

^i^ Calculation based on women 20–29 years.

^j^ Calculation based on women 30–39 years.
