# Supplementary material for: Characteristics of women obtaining induced abortions in selected low- and middle-income countries
Source: PLoS One. 2017 Mar 29;12(3):e0172976. doi: 10.1371/journal.pone.0172976 (PMC5371299; doi:10.1371/journal.pone.0172976)
Supplement: S1 Table — (PDF) [file pone.0172976.s001.pdf]

S1 Table. List of sources of data.

Region/Country	Coverage	Data	Year	Data Source
Africa				
Congo Republic	Nationally representative	Population-based	2011-12	Demographic and Health Survey
Ethiopia	Nationally representative of abortions obtained in public and private sector health facilities. Abortions in NGO affiliated facilities are not included.	Abortion patient	2014	Prospective Data Survey
Gabon	Nationally representative	Population-based	2012	Demographic and Health Survey
Ghana	Nationally representative	Population-based	2007	Maternal Health Survey
Nigeria	Eight states (covering all four health zones)	Population-based	2002-03	Community-based Survey
Asia				
Armenia	Nationally representative	Population-based	2010	Demographic and Health Survey
Azerbaijan	Nationally representative	Population-based	2006	Demographic and Health Survey
Bangladesh	Nationally representative	Population-based	2011	Demographic and Health Survey
Cambodia	Nationally representative	Population-based	2010	Demographic and Health Survey
Georgia	Nationally representative	Official Statistics	2011	UN Demographic Yearbook
Kyrgyz Republic	Nationally representative	Population-based	2012	Demographic and Health Survey
Nepal	Nationally representative	Population-based	2011	Demographic and Health Survey
Pakistan	Nationally representative	Population-based	2012-13	Demographic and Health Survey
Philippines	Nationally representative	Population-based	2004	Community-based Survey
Tajikistan	Nationally representative	Population-based	2012	Demographic and Health Survey
Turkey	Nationally representative	Population-based	2008	Demographic and Health Survey
Uzbekistan	Nationally representative	Population-based	2002	Health Examination Survey
Vietnam	Nationally representative	Population-based	2002	Demographic and Health Survey
Europe				
Albania	Nationally representative	Population-based	2008-09	Demographic and Health Survey
Belarus	Nationally representative	Official Statistics	2013	UN Demographic Yearbook
Bulgaria	Nationally representative	Official Statistics	2013	UN Demographic Yearbook
Moldova	Nationally representative	Population-based	2005	Demographic and Health Survey
Montenegro	Nationally representative	Official Statistics	2005	UN Demographic Yearbook
Romania	Nationally representative	Official Statistics	2012	UN Demographic Yearbook
Serbia	Nationally representative	Official Statistics	2008	UN Demographic Yearbook
Ukraine	Nationally representative	Population-based	2007	Demographic and Health Survey
Central America and the Caribbean				
Haiti	Nationally representative	Population-based	2012	Demographic and Health Survey
Mexico City (Mexico)	City (Mexico City). Representative of abortions obtained in public sector facilities. Abortions obtained in private sector facilities are not included.	Abortion patient	2007-2010	Mondragon y Kalb et al., Patient characteristics and service trends following abortion legalization in Mexico City, 2007-10. Studies in Family Planning, Vol. 42, No.3, pp. 159-166.
