# Supplementary material for: Characteristics of women obtaining induced abortions in selected low- and middle-income countries
Source: PLoS One. 2017 Mar 29;12(3):e0172976. doi: 10.1371/journal.pone.0172976 (PMC5371299; doi:10.1371/journal.pone.0172976)
Supplement: S2 Table — (PDF) [file pone.0172976.s002.pdf]

S2 Table. Characteristics of women obtaining abortions in Ethiopia and Nigeria by data source.

	Ethiopia 2014		Nigeria 2003[a]	
	Abortion patient survey	Post-abortion patient survey	Population-based survey	Post-abortion patient survey
Age (%)				
15-19	28.7	10.7	34.2	17.3
20-24	36.8	26.3	19.7	32.1
25-29	22.7	27.7	21.2	22.0
30-34	7.9	20.1	11.0	16.1
35-39	3.3	11.2	9.6	8.0
40-44	0.5	4.1	4.2	4.7
Marital Status (%)				
Married	40.9	85.4	41.5	52.0
Unmarried	59.1	14.6	58.5	48.0
Parity (%)				
Zero	NA	NA	49.9	51.6
One	NA	NA	13.1	11.4
Two or more	NA	NA	37.0	37.1
Education (%)				
None or primary	53.1	74.4	38.3	23.0
Secondary or more	46.9	25.6	61.8	75.2
Residence (%)				
Urban	NA	NA	35.3	82.0
Rural	NA	NA	64.7	18.0
N	2,706	2,898	205	2,336

Note: Percentages are presented as proportion of non-missing responses. NA = Data are not available.

[a] Population-based survey covers both urban and rural areas. Post-abortion patient survey is restricted to urban areas.
