# Supplementary material for: Characteristics of women obtaining induced abortions in selected low- and middle-income countries
Source: PLoS One. 2017 Mar 29;12(3):e0172976. doi: 10.1371/journal.pone.0172976 (PMC5371299; doi:10.1371/journal.pone.0172976)
Supplement: S3 Table — (PDF) [file pone.0172976.s003.pdf]

S3 Table. Abortion rates (number of abortions per 1000 women ages 15-44) by country in Africa, Europe, and Central America and the Caribbean.

	Congo Republic[a]	Gabon[a]	Ghana	Nigeria[a]	Albania	Belarus[b]	Bulgaria[b]	Moldova	Montenegro[b]	Romania[b]	Serbia[b]	Ukraine	Haiti[a]
Age													
15-19	29.6	22.7	17.2	34.3	2.2	7.6	15.4	8.4	2.3	14.5	4.6	6.3	3.5
20-24	48.4	38.5	24.7	19.4	9.7	17.8	26.4	55.6	14.3[d]	25.2	14.2	18.2	8.4
25-29	36.1	34.4	15.9	21.1	15.5	22.0	30.7	59.4	-	25.3	20.3	26.3	8.8
30-34	25.2	26.4	11.1	16.0	14.9	22.2	27.4	53.7	23.1[e]	23.2	23.4	18.9	7.1
35-39	18.1	17.6	9.4	17.3	8.8	14.1	19.6	35.5	-	17.8	20.5	11.8	7.6
40-44	8.1	10.3	3.5	12.3	3.8	6.7	6.5	9.1	10.3	6.7	10.1	5.3	3.7
Marital Status[c]													
Married	28.7	27.0	11.9	12.6	11.7			44.2				18.1	7.7
Unmarried	34.1	27.1	20.3	42.6	1.3			13.6				9.3	4.8
Parity													
0	26.4	20.9	19.0	38.5	1.2			12.2				5.9	4.3
1	41.8	39.7	13.8	21.7	7.0			58.8				18.2	10.0
2+	28.8	26.0	12.7	13.6	3.7			41.6				21.4	7.2
Wealth[c]													
Poorest	15.1	28.3	6.8	13.7	7.4			22.2				22.1	0.5
Second	30.6	30.2	5.9	24.3	9.1			36.0				17.8	1.5
Middle	34.7	26.7	15.9	19.2	11.5			30.2				15.4	5.0
Fourth	35.1	27.0	20.0	30.1	8.4			35.5				11.8	9.9
Richest	34.7	24.1	21.5	31.2	5.6			44.5				10.9	10.8
Education[c]													
Less than secondary	26.7	19.6	9.5	13.1	9.8			95.9				NA	5.9
Secondary or more	32.5	29.5	19.8	35.9	6.9			34.1				14.7	7.0
Residence[c]													
Urban	35.7	27.1	20.6	22.8	8.3			39.4				13.0	9.2
Rural	19.8	26.8	10.5	21.1	8.4			30.8				19.2	3.9
Total Abortion Rate	0.8	0.7	0.4	0.6	0.3	0.5	0.6	1.1	0.4	0.6	0.5	0.4	0.2
General Abortion Rate	30.8	27.1	14.9	21.7	8.4	15.6	21.1	34.6	14.3	18.6	15.8	14.7	6.5

Note: All data are from population-based surveys. Calculations are based on all abortions reported in the three year period before the survey unless otherwise noted. Abortion rates are most likely underestimates due to underreporting of abortions. Abortion rates for Ethiopia and Mexico are not presented because data are from an abortion patient survey. NA = Not applicable.

[a] Calculations based on the most recent reported abortion in the three year period before the survey.

[b] Data are from official statistics on legal abortion.

[c] Characteristic measured at the time of the survey.

[d] Calculation based on women 20-29 years.

[e] Calculation based on women 30-39 years.
