# Supplementary material for: Characteristics of women obtaining induced abortions in selected low- and middle-income countries
Source: PLoS One. 2017 Mar 29;12(3):e0172976. doi: 10.1371/journal.pone.0172976 (PMC5371299; doi:10.1371/journal.pone.0172976)
Supplement: S4 Table — (PDF) [file pone.0172976.s004.pdf]

S4 Table. Abortion rates (number of abortions per 1000 women ages 15-44) by country in Asia.

	Armenia	Azerbaijan	Bangladesh[a]	Cambodia[b]	Georgia[c]	Kyrgyz Republic	Nepal	Pakistan	Philippines	Tajikistan	Turkey	Uzbekistan	Vietnam
Age[d]													
15-19	16.0	30.9	3.2	9.1	11.2	4.0	5.3	2.3	16.0	2.3	8.2	4.9	1.2
20-24	56.0	119.1	7.1	16.5	42.2[e]	28.9	10.5	2.5	22.3	14.3	12.5	32.1	22.6
25-29	83.6	176.4	11.9	18.3	-	49.1	18.8	5.4	22.1	25.1	21.8	50.7	40.3
30-34	54.5	156.2	11.2	27.8	52.1	34.5	16.1	6.3	18.1	26.4	26.4	52.3	31.1
35-39	22.0	93.7	9.3	26.8	24.7	26.5	8.9	2.7	17.0	28.9	28.0	40.9	32.1
40-44	11.8	39.1	5.7	16.9	4.1[f]	13.9	2.0	2.2	13.5	11.8	14.2	28.6	19.5
Parity[g]													
0	6.4	6.8	5.8	8.0		4.7	22.4	2.1	27.3	3.7	8.9	6.9	4.4
1	40.3	104.3	7.7	15.5		29.6	15.3	3.8	22.3	14.9	19.7	38.5	23.2
≥2	54.0	135.0	8.6	22.9		36.7	6.0	4.4	17.4	26.5	23.4	46.0	34.1
Wealth[h]													
Lowest	56.4	141.9	4.9	17.6		21.2	7.0	0.8	15.7	17.3	14.3		31.1
Second	35.8	85.8	6.8	18.2		25.3	7.8	2.4	-	14.0	20.8		25.1
Middle	57.6	117.5	6.6	18.1		21.9	8.6	3.5	19.7	16.9	21.5		31.1
Fourth	34.6	92.9	8.8	26.2		38.4	12.2	5.3	-	21.4	14.1		31.1
Richest	35.7	97.5	13.0	21.9		39.7	19.6	7.2	22.0	25.2	29.7		24.9
Education[h]													
Less than secondary	67.9	120.5	6.0	21.5		-	8.0	3.4	13.8	8.6	20.5	25.0	26.0
Secondary or more	43.2	106.7	10.9	17.2		29.5	17.5	5.0	21.1	19.7	20.1	39.7	30.0
Residence[h]													
Urban	45.1	109.9	12.2	25.2		39.4	16.1	5.4	18.6	25.7	21.0	48.8	28.9
Rural	43.3	103.4	6.8	19.4		24.6	10.5	3.1	18.8	16.9	18.3	32.2	28.6
Total Abortion Rate	1.2	3.1	0.2	0.6	0.9[i]	0.8	0.3	0.1	0.5	0.5	0.6	0.9	0.7
General Abortion Rate	44.3	106.9	8.2	20.4	26.4[i]	29.4	11.3	3.9	18.7	19.0	20.4	38.5	28.7

Note: All data are from population-based surveys unless otherwise specified. Calculations are based on currently married women unless otherwise noted and all abortions reported in the three year period before the survey. Abortion rates are most likely underestimates due to underreporting of abortions.

[a] Menstrual regulation used as proxy for abortion.

[b] Calculations based on reported abortions in the five year period before the survey.

[c] Calculations based on abortions obtained in the year of data collection and all women, regardless of marital status. Data are from official statistics on legal abortion.

[d] For Bangladesh and Cambodia, calculations based on age at the time of survey.

[e] Calculations based on women 20-29 yrs.

[f] Calculations based on women 40-49 yrs.

[g] For Philippines, parity measured at the time of the survey.

[h] Characteristic measured at the time of the survey.

[i] Calculations based on women 15-49 yrs.
