# Supplementary material for: Characteristics of women obtaining induced abortions in selected low- and middle-income countries
Source: PLoS One. 2017 Mar 29;12(3):e0172976. doi: 10.1371/journal.pone.0172976 (PMC5371299; doi:10.1371/journal.pone.0172976)
Supplement: S5 Table — (PDF) [file pone.0172976.s005.pdf]

S5 Table. Percentage distribution of reproductive-age women by sociodemographic characteristics, by region and country.

Region/Country	Age group						Married	Parity		
	15-19	20-24	25-29	30-34	35-39	40-44		0	1	2+
Africa										
Congo Republic	21.8	20.1	20.0	15.5	13.5	9.1	57.4	25.0	19.1	55.9
Ethiopia[a]	26.2	19.1	20.5	13.4	12.5	8.2	61.3	NA	NA	NA
Gabon	22.7	20.9	18.9	15.4	12.6	9.5	52.3	31.1	20.4	48.5
Ghana	22.0	18.7	17.8	16.0	14.9	10.6	58.4	32.8	15.3	51.9
Nigeria[b]	24.4	21.2	19.6	13.4	11.6	9.8	68.7	33.3	13.5	53.2
Asia [c]										
Armenia	2.3	15.3	21.8	22.1	17.6	20.9	NA	8.8	19.5	71.7
Azerbaijan	3.4	15.5	17.9	18.4	20.6	24.3	NA	10.1	15.7	74.2
Bangladesh	12.7	22.4	21.6	16.7	13.8	12.8	NA	10.4	22.2	67.4
Cambodia	3.8	16.7	25.6	18.0	17.4	18.5	NA	7.8	21.3	70.8
Georgia[d]	13.1	30.7[g]	-	14.1	13.7	28.3[h]	NA	NA	NA	NA
Kyrgyz Republic	3.5	19.7	23.4	19.1	17.6	16.7	NA	9.4	19.7	70.9
Nepal	9.0	20.1	21.8	18.9	16.6	13.6	NA	11.5	18.7	69.9
Pakistan	5.2	17.8	23.1	21.3	18.6	14.0	NA	13.8	13.5	72.7
Philippines[e]	3.2	14.6	20.6	22.1	21.7	17.8	NA	8.5	19.2	72.3
Tajikistan	4.6	23.0	23.2	17.7	16.1	15.3	NA	12.2	16.2	71.6
Turkey	3.0	13.7	21.9	22.1	21.0	18.3	NA	10.1	22.1	67.9
Uzbekistan	2.4	20.9	21.8	20.0	18.2	16.7	NA	7.9	14.8	77.3
Vietnam	1.5	11.5	21.0	22.8	22.4	20.8	NA	5.3	20.7	74.0
Europe										
Albania	22.8	15.0	13.1	13.3	16.9	19.0	61.7	41.7	9.6	48.8
Belarus	12.3	17.0	19.2	17.8	16.9	16.9	NA	NA	NA	NA
Bulgaria	12.0	16.2	16.8	17.6	19.2	18.2	NA	NA	NA	NA
Moldova	22.5	17.9	15.3	14.7	13.6	16.0	63.3	37.9	22.7	39.4
Montenegro	17.4	35.2[g]	-	31.2[i]	-	16.2	NA	j	j	j
Romania	11.7	15.9	16.9	17.9	17.6	20.0	NA	NA	NA	NA
Serbia	14.7	16.7	17.4	17.7	16.7	16.8	NA	NA	NA	NA
Ukraine	13.6	17.5	17.4	17.1	18.2	16.3	58.5	35.3	36.4	28.4
Central America and the Caribbean										
Haiti	25.5	21.7	18.3	13.9	11.3	9.3	52.9	43.3	18.4	38.3
Mexico City[f]	15.7	16.7	17.4	17.5	17.0	15.7	NA	NA	NA	NA

Note: All sociodemographic characteristics are taken from the same data source and year as abortion data unless otherwise noted. NA = Not applicable.

[a] Sociodemographic characteristics are taken from the 2011 Ethiopia DHS.

[b] Sociodemographic characteristics are taken from the 2003 Nigeria DHS.

[c] Calculations based on samples of currently married women unless otherwise specified.

[d] Calculations based on all women. Sociodemographic characteristics are averaged from the 2010 and 2012 Demographic Yearbook.

[e] Sociodemographic characteristics are taken from the 2003 Philippines DHS.

[f] Sociodemographic characteristics taken from CONAPO, Proyecciones de la población de México 2005–2050, <http://www.conapo.gob.mx/es/CONAPO/De_la_poblacion_de_Mexico_2005-2050>, accessed Nov. 15, 2012.

[g] Calculation based on women 20-29 years.

[h] Calculation based on women 40-49 years.

[i] Calculation based on women 30-39 years.

[j] No available data on parity. Total fertility rate (TFR) = 1.8 births per woman. TFR data are taken from the World Bank World Development Indicators.
