# Supplementary material for: Characteristics of women obtaining induced abortions in selected low- and middle-income countries
Source: PLoS One. 2017 Mar 29;12(3):e0172976. doi: 10.1371/journal.pone.0172976 (PMC5371299; doi:10.1371/journal.pone.0172976)
Supplement: S6 Table — (PDF) [file pone.0172976.s006.pdf]

S6 Table. Percentage distribution of abortions by age and data source.

Country, Year	Data source	15-19	20-24	25-29	30-34	35-39	40-44	N
Azerbaijan, 2006	DHS	1.8	18.7	27.1	26.5	17.8	8.0	1,504
Azerbaijan, 2007	DY	4.9	22.1	31.3	24.4	17.3[a]	-	22,323
Kyrgyz Republic, 2012	DHS	1.1	21.7	34.4	21.1	14.0	7.7	427
Kyrgyz Republic, 2008	DY	8.7	24.1	26.3	22.1	13.3	5.4	20,800
Tajikistan, 2012	DHS	1.3	17.4	26.8	22.3	23.2	9.0	365
Tajikistan, 2012	DY	7.3	69.6[b]	-	-	23.1[a]	-	16,618
Moldova, 2005	DHS	5.3	27.0	26.2	22.5	14.2	4.6	607
Moldova, 2006	DY	9.1	75.5[b]	-	-	15.4[a]	-	15,742
Ukraine, 2007	DHS	6.1	21.4	31.2	21.4	13.6	6.2	278
Ukraine, 2007	DY	9.4	73.8[b]	-	-	16.8[a]	-	210,454

[a] Calculation based on women 35+ years.

[b] Calculation based on women 20-34 years.
